# Shared Accountability Shaping the Destinies of Individual and Groups of Nonhuman Primates

**DOI:** 10.3390/vetsci11100486

**Published:** 2024-10-09

**Authors:** Ori Pomerantz, Gregory Brion Timmel

**Affiliations:** California National Primate Research Center, University of California, Davis, CA 95616, USA; gbtimmel@ucdavis.edu

**Keywords:** rhesus macaque, group housing, collaboration, welfare, integrative approach

## Abstract

**Simple Summary:**

At the California National Primate Research Center (CNPRC), rhesus macaques are kept in large, complex social groups outdoors because this environment helps them behave naturally and cope well. However, living in groups can lead to problems, such as fights that sometimes result in injuries. To handle these situations, a team of experts from different fields regularly meets to discuss and decide if any macaques need to be moved to protect them from repeated attacks. The team uses a careful process to identify which animals might be at risk and decide whether the individual should stay in the social group or be relocated, keeping in mind both the animal’s well-being and the stability of the whole group. This paper includes real-life examples to show how they make these tough decisions, aiming to keep all the animals physically and mentally healthy while maintaining harmony in the group.

**Abstract:**

At the California National Primate Research Center (CNPRC), the preferred housing for rhesus macaques involves maintaining them in complex social groups outdoors, primarily for breeding purposes. This functionally appropriate environment promotes effective coping through the expression of species-typical behaviors and important aspects of species-typical social structure, thus enabling normal animal development, higher reproductive success, and the production of high-quality biological models. Despite the benefits, social housing introduces challenges like trauma from aggressive interactions. These challenges necessitate a delicate balance between tolerating some aggression and preventing repeated targeting of individuals. Therefore, the CNPRC has established a multidisciplinary working group of behavioral management experts, veterinarians, animal care, and researchers that meets regularly to review cases of animals that may need to be removed from their social group. We discuss the criteria and decision-making processes employed to manage and mitigate aggression. We describe the systematic approach to identifying at-risk individuals and the comprehensive evaluation process that guides whether to relocate an animal from their groups or not. Considerations include the welfare of the individual and the group’s social stability. This paper provides case studies demonstrating how the working group applies these criteria and processes in practical scenarios, highlighting the complexities and challenges of such decisions.

## 1. Introduction

Providing excellent quality care for nonhuman primates (NHPs) is of the utmost importance for the faculty and staff of the California National Primate Research Center (CNPRC). While regulatory requirements of care are abundant (e.g., [1,2]), faculty and staff are motivated to go above and beyond these standards to support the animals’ needs. To achieve this goal, we integrate behavioral and clinical expertise to ensure optimal outcomes for the NHPs and the research they contribute to. Inadequacy in addressing the animals’ needs can affect their psychological and physiological functioning and, consequently, their welfare and the ability to provide reliable scientific data. Conversely, proper animal management that successfully addresses the many aspects of the animals’ needs promotes their welfare and contribution to biomedical research. Providing appropriate responses to a wide variety of (psychological and physiological) aspects of the NHPs’ biological needs requires a wide variety of expertise. In this paper, we describe how bringing together diverse expertise in behavioral management and veterinary medicine enables us to reach our goal of excellent care.

Macaques (*Macaca* spp.) naturally live in complex, multi-male, multi-female, and inter-generational social groups [3]. Rhesus macaques (*Macaca mulatta*) form female-bonded societies (female philopatry), whereby males disperse from their natal group around the age of puberty, and females remain in the groups they are born into (and develop life-long relationships with other females) [4]. Kinship has been suggested to be a central stabilizing factor in rhesus macaque social groups. Beisner et al. [5] have shown that the more animals are genetically related, the less likely they are to engage in severe aggression and inflict wounding on one another. Group living is advantageous since it protects group members against predators [6] and rival conspecifics [7] and, consequently, their fitness. NHPs maintain normal group function by expressing a rich repertoire of social behaviors [8], such as allogrooming and post-conflict reconciliation, to uphold the group’s cohesiveness and to regulate destabilizing aggressive interactions that put the animals at risk [9,10]. Social behaviors are, therefore, crucial in contributing to group members’ ability to survive and reproduce. NHPs are motivated to express behaviors that significantly impact their fitness [11], likely due to the rewarding sensation accompanying their expression [12,13,14]. A chronic inability to express specific motivated behaviors can induce frustration and lower the animal’s welfare [13,15]. For example, Pomerantz et al. [16] suggested that NHP species may develop abnormal hair-plucking when thwarted from expressing social grooming. Therefore, it is considered best practice to house NHPs, including rhesus macaques, in a physically and socially complex environment that functionally resembles their natural habitat and enables the normal expression of social behaviors [17,18,19].

The CNPRC’s outdoor social groups serve two major purposes: breeding and research. Most animals that serve a research purpose are removed from the group and brought to a more controlled indoor environment where they can be readily accessed. However, some studies require the animals to be housed in social groups to be able to test their hypotheses. Examples include research on social networks [20], disease transmission [21], and the effects of wildfires on respiratory function [22].

Research has shown that environments that enable complex social interactions benefit NHPs (e.g., [19]). At the CNPRC, the highest degree of social complexity is observed in large breeding groups. Living in social groups provided some protection for rhesus macaques from developing stereotypic and self-injurious behavior (SIB) compared to singly housed or pair-housed monkeys [23], and those abnormal behaviors were significantly reduced following their relocation to large social groups [24]. However, while living in a social group generally benefits the animals, it also has its costs. Living in a social group involves competition over access to resources, which may result in trauma due to aggressive interactions with group members [25]. In the wild, NHPs can remove themselves from a conflict by fleeing from the aggressor, and, if fleeing is not possible, they may employ an array of submissive behaviors to diffuse the situation [26]. In captivity, fleeing options are reduced compared to the wild, in a manner that negatively impacts the animal’s capability to cope successfully with the aggressive encounter [27]. Modern enclosure design needs to consider escape routes and additional means, such as visual barriers, as measures to prevent or alleviate intragroup aggression [28]. The use of bedding material in enclosures to modify foraging conditions was additionally found to reduce aggression [29]. However, despite the use of such environmental features, social aggression cannot be eliminated from captive rhesus macaque societies. In fact, as a highly despotic species, a lesser degree of aggression is expected and should be tolerated since it is involved in conflict regulation [19,30]. Moreover, male rhesus macaques seem to inhibit aggression directed toward helpless victims [31], and similar forms of self-restraint have also been reported [32]. On the other hand, repeated targeting of individuals by other group members should be carefully assessed, particularly if it is determined that the targeted individual is likely to be a victim of aggression again or if the aggressive encounter was severe. In such cases, animals (either the target of aggression or, preferably, the instigator of the aggression) will likely need to be removed from the group [33]. Relocating individuals from their social group affects not only the relocated group member but also has the potential to change group dynamics and stability [34]. Such instability is characterized by increased agonistic interactions, social trauma, and even mortality, and this is particularly evident when the removed individuals routinely engage in conflict regulation of conspecifics [34] and when key social figures like the alpha male or female are absent [35]. Removal of non-alpha matriarchs from their social group appeared to have a comparable effect on social stability [36]. Finally, removing animals that support their kin can also trigger social instability. Relocating the daughters of an alpha female encouraged females from other matrilines to overthrow the presiding leadership [37]. It is, therefore, clear that since the removal of particular individuals may affect the social stability of the group, one must consider the welfare of the individual and the entire group.

Removed individuals are brought indoors to a transition or hospital room and placed in standard stainless-steel cages, according to [1,2], for a minimum of two weeks, during which they undergo daily health assessments. If no clinical issues are noted (e.g., diarrhea), the animals are relocated to a non-transitional room where they may await assignment to research projects, future group formation, or shipment to other facilities. Whenever possible, animals are pair-housed with a compatible partner upon their arrival indoors. However, a veterinarian may exempt an animal from being socially housed if its clinical condition warrants that. Similarly, certain screening procedures for research projects may also require single housing, and the behavioral management staff may avoid beginning social introductions of animals slated for imminent studies that require single housing. Acknowledging the significant change that animals experience when moved to a more confined and less socially and structurally complex living environment and the potentially negative effects it may have on them, we have recently begun implementing a transitioning plan to help those removed animals adapt to the changes. The plan focuses on strengthening the bonds between the animals and their care staff by initiating both unstructured (i.e., play and grooming) and structured (i.e., positive reinforcement training) positive human–animal interactions, prioritizing animals for social housing, and providing additional environmental enrichment and visual barriers such as privacy panels when needed. Anecdotal information from animal care staff suggests that such early interventions decrease the animals’ degree of arousal. We plan on conducting a thorough scientific evaluation to assess the effects of such interventions on the animals’ adaptiveness to their new environment.

Rhesus macaques are seasonal breeders with a typical breeding season (fall–winter) and a birthing season (spring–summer) [38]. Approximately 700–800 offspring are born into the CNPRC breeding colony yearly. Those monkeys then remain in the group, where they grow up and contribute to the reproductive pool or are assigned to a research project (which may or may not require permanent relocation indoors). In the aftermath of the COVID-19 pandemic, it became clear that there was a shortage of available NHPs for research [39]. All NHP breeding facilities were called upon to address this macaque shortage and maximize breeding success. In doing so, breeding facilities needed to be mindful of the stressful effects of social instability on breeding success. Indeed, the interplay between the hypothalamic–pituitary–adrenal (HPA) and the hypothalamic–pituitary–gonadal (HPG) axes has been studied extensively [40]. The stress experienced by animals in an uncertain social environment can have an inhibitory effect on the physiological and behavioral functioning of their reproductive system [41]. In chimpanzees (*Pan troglodytes*), perturbations to group stability due to neighbor pressure have led to lower rates of offspring survival and longer inter-birth intervals [42]. Similarly, Dettmer et al. [37] reported that a matrilineal overthrow in a rhesus macaque group resulted in 72% infant mortality (the highest measured in this group), half of which were due to miscarriages that occurred shortly after the overthrow. The authors additionally highlighted the long-term effects of the matrilineal overthrow, in that the attacked matriline did not reproduce viable offspring for three years following the overthrow. It is, thus, not surprising that reproductive dysfunction is often employed as an indicator of poor welfare [43] (however, the inability to reproduce and raise offspring does not necessarily induce poor welfare (e.g., [44])). This situation further highlighted the importance of maintaining normally functioning social groups that breed and add healthy animals that can serve as high-quality subjects in translational biomedical research.

**Figure 1:**
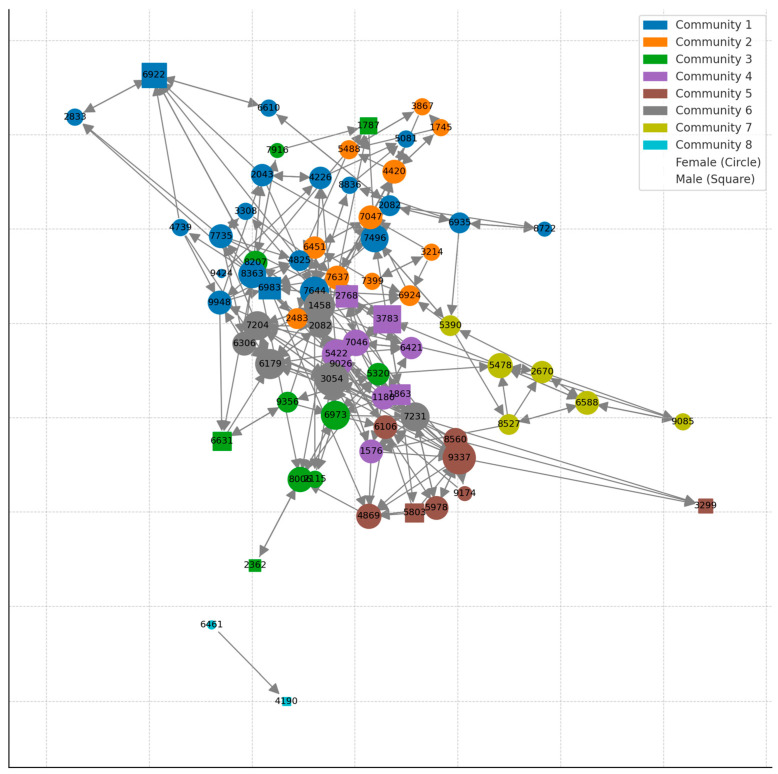
An example of a grooming network subjected to a community detection analysis (Louvain Method—Greedy Modularity Maximization). The results are plotted as a directed network graph where color is community, shape is sex, and size of the node is degree. Similar networks are used to identify the level of social integration of individuals and the likelihood impact of their removal on the social stability of the group. Figure 1 was generously provided by Brenda McCowan.

Each group is systematically and methodically observed by experienced behavioral management staff members who use the collected data to construct social hierarchies and networks that affect management decisions (Figure 1). These networks can help identify more socially connected animals and are thus more likely to affect the group’s social organization if removed. For example, in Figure 1, animals 3054 (gray) and 9337 (brown) have more social partners with whom they groom, whereas animal 2362 (green) engages in allogrooming with just one animal. It is, therefore, likely that removing 2362 from the group will be less disruptive to the group’s stability than 3054 and 9337.

**Figure 2:**
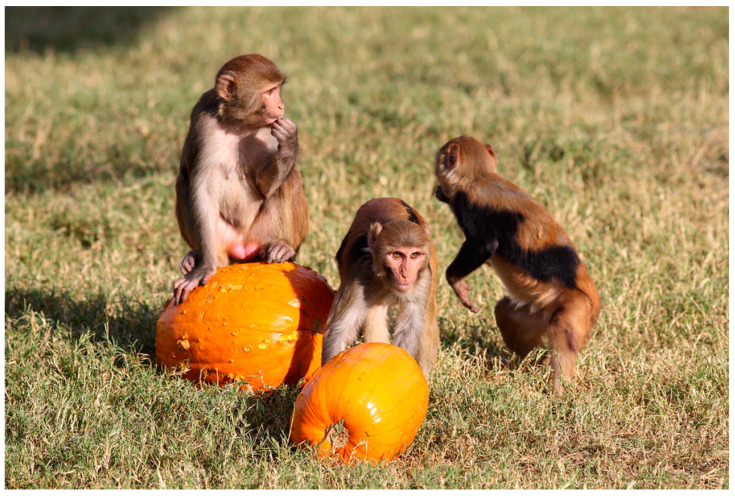
Provisioning of long-lasting produce was found to reduce socially inflicted trauma in large groups of rhesus macaques (Photo credit—Kathy West).

**Figure 3:**
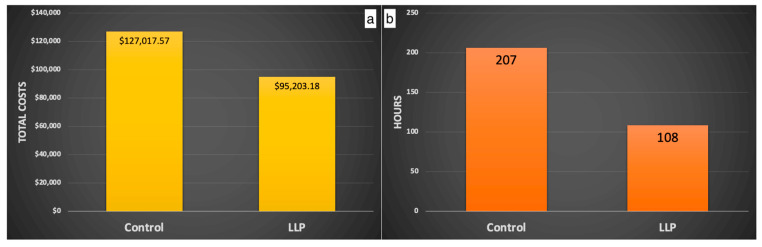
Using long-lasting produce (LLP) results in lower costs (**a**) and time (**b**) associated with veterinary treatment for socially inflicted trauma during the breeding season. Copied with permission from McCowan et al., 2024, unpublished data.

In addition, animal care staff conduct bi-daily health assessments to spot signs of illness, injury, or unusual behavior, utilizing their familiarity with each individual animal to recognize actions that deviate from the animal’s typical behavior patterns. Animals presenting such signs are considered for removal from the enclosure by the animal care and clinical staff. Those removed are delivered to the hospital to be examined by the veterinary staff. Behavioral management staff are notified when the removed animals are alpha and beta males and females; when animals sustain severe trauma (i.e., crush/multiple lacerations); when several animals from the same enclosure go to the hospital with trauma; and when more than two animals have facial trauma in an enclosure. These notifications alert the behavioral management staff of an increased risk of social destabilization and trauma and assess if additional manipulations are needed to prevent social perturbations. Manipulations may include the temporary or permanent removal of additional group members to maintain the social equilibrium, enhanced provision of enrichment items such as long-lasting produce that effectively reduces trauma and associated costs ([45]—Figure 2 and Figure 3), and increased behavioral monitoring of the group. Group stability is subsequently evaluated primarily by trauma rates and dominance certainty (a measure of rank ambiguity) [46].

Social instability in large outdoor groups is alarming for several reasons. First, there are obvious concerns about the health and welfare of injured animals. Second, it can negatively impact breeding success (especially when a full matrilineal overthrow occurs) [37]. Third, disruptions to ongoing research conducted outdoors are likely, and logistic challenges are associated with the need to relocate a large number of animals indoors (either to be treated in the hospital or following group disbandment). There may be rare instances in which the welfare of the individual would appear at odds with the study’s goals. For example, behavioral and clinical staff may advocate for the permanent relocation of an animal that has been repeatedly the victim of aggression and would likely be targeted again if it remained in the group. The same animal, however, may be providing important data to the study to which it is assigned. Therefore, removing the animal from the group could potentially result in a loss of crucial data. In these rare cases, the welfare of the animal always trumps the research needs. Veterinary and behavioral management staff work closely with the investigator to devise a plan that prioritizes the health and welfare of the individual animal while minimizing losses and interferences to the research. Fourth, veterinary treatment of trauma is costly since it requires a significant time investment to treat the animals clinically and resources that could otherwise be used to benefit other animals. Indeed, McCowan et al. [30] demonstrated that the risk of injury is so prevalent that a significant proportion of the animals in a breeding group (up to 60%) may be hospitalized within a year, the cost of which is estimated in the hundreds of thousands of U.S. dollars a year [45].

In light of the wide impact of social instability on the CNPRC’s operations and the need to make informed decisions that consider all stakeholders, we have created a working group that consists of behavioral management experts, investigators, veterinarians, and animal care staff. The group’s mission is to gather pertinent information regarding individual animals that have been the target of aggression and, based on the information provided by the different departments, determine whether relocating an animal from its social group best serves the individual and the group.

## 2. Materials and Methods

### 2.1. The Working Group

The working group meets every two weeks, either in person or remotely. A list of candidates for relocation is distributed prior to the meeting to allow members to gather pertinent information before the planned discussion. The length of each meeting depends on the number of animals on the agenda and the complexity of their cases. Every case is discussed individually, and a decision is finally made. The ultimate decision belongs to the veterinarians for any clinical case. However, any socially driven relocations that do not have any clinical concerns are generally left up to the behavioral management staff.

### 2.2. The Review Process

#### 2.2.1. Criteria to Identify Candidate Animals for Permanent Removal from Their Social Group

The first step of the review process is to identify potential candidates for removal from their group. The working group will review individuals if any of the following conditions apply:

1. Animals with severe trauma that resulted in abnormal physical use of the affected area.
2. Animals that sustained multiple-digit traumas requiring amputation at a single trauma event.
3. Animals presented with lacerations to one or more muscles totaling 5 cm or more.
4. Presentation for trauma 0–3 days after hospital discharge for trauma.
5. Three or more presentations of trauma within 12 months, each requiring >2 regular workdays of hospitalization.

If cases do not fall within the criteria listed above, veterinary staff may contact the behavioral management staff to discuss the potential for removal from the group.

#### 2.2.2. Criteria to Determine Whether Animals Should Be Permanently Removed from Their Social Group

At the meeting, preidentified cases are reviewed individually by the working group using the following set of questions that were developed to ensure consistency in the review process:

1. Did group members mob the animal?
2. Would the dynamics of the group be significantly affected if the animal were to be relocated?
3. Was the animal assigned to a research project conducted outdoors?
4. How frequently does the animal sustain socially inflicted trauma?
5. What was the duration between the trauma events?
6. What was the severity of the injuries (number of wounds, depth of wounds, muscle involvement, the extent of treatment prior to the resolution of injury, partial or complete loss of function)?
7. What are the long-term health and welfare consequences of repeat injury or condition (e.g., the loss of multiple digits in a young animal may make future injury more significant, and significant scar tissue or muscle deficit may make repeated injury more substantial)?
8. Can the animal maintain good health outdoors?

At the conclusion of the review, a final decision is made (e.g., relocation indoors, discharge back to home enclosure, pending removal of the aggressing animal(s), discharge to the home enclosure with increased monitoring by behavioral management staff, and discharge to the home enclosure with later relocation if the animal presents again for trauma in a designated length of time). Finally, an entry summarizing that decision is generated and added to the animal’s medical record. It should be noted that some situations are severe enough to warrant an automatic permanent removal from the group. These situations involve any crushing trauma to multiple extremities, severe crushing trauma to one or more body regions, and a permanent loss of function.

### 2.3. Socio-Environmental Characteristics of the Outdoor and Indoor Environments

Relocation from a social group almost always involves transitioning the animal from an outdoor (where most social groups live) to an indoor (where animals can be more easily accessed) environment. Relocated animals are then housed in standard stainless-steel caging per regulatory requirements [1,2]. Once cleared by the veterinary staff, these animals can be socially housed with a compatible partner or remain singly housed if clinically or scientifically mandated (e.g., if screening for a research project requires that). Both the frequency and duration of removal from a social group vary substantially. For example, removals due to trauma are more frequent during the breeding season, but the duration of treatment and the decision of whether permanent relocation indoors is required is determined by factors such as the severity and type of trauma. In addition, animals may be removed for screening prior to research assignments or shipments, and those instances are affected by the onset of research studies and the demand for animal sales. Finally, behavioral management staff may temporarily relocate individuals to manipulate the social environment (e.g., relocating animals that may be targeted by other group members when their “protector” is out due to trauma). The duration of these types of removals is as short as possible and is dependent on the ability of the “protector” to return to the group. Here, we describe the two distinct environments at our facility.

#### 2.3.1. The Outdoor Environment

**Figure 4:**
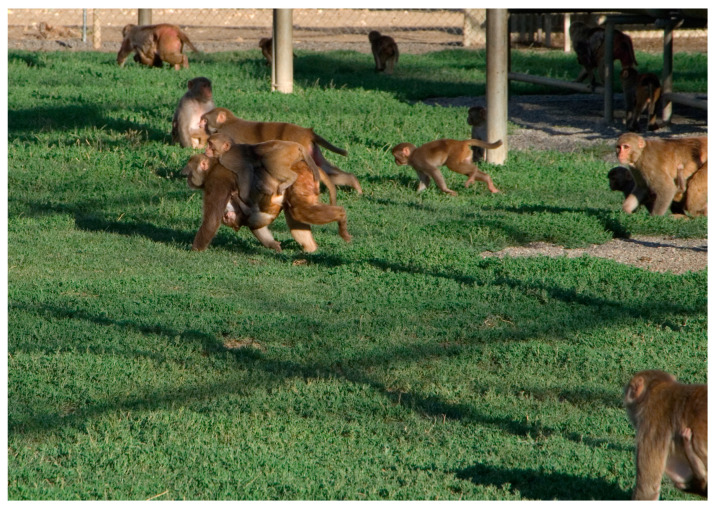
An example of a social group of rhesus macaques at the CNPRC.

We house most animals in twenty-four 0.20 ha outdoor corals, with multi-male, multi-female groups of approximately 50–200 individual rhesus macaques each (Figure 4). Every enclosure has a variety of structural enrichment items (i.e., cage furniture), visual barriers, and toys and manipulanda. The animals received commercial monkey chow (LabDiet 5047 High Protein Monkey Diet Jumbo, Hayward, CA, USA) supplemented by nuts, seeds, and grains to encourage foraging behavior. The animals additionally received fresh produce 1–2 times per week. Water was provided ad libitum.

#### 2.3.2. The Indoor Environment

The majority of the indoor macaques are housed as pairs with continuous full access to each other. Smaller portions are housed alone in a cage (within sight and sound of conspecifics and with proper scientific, clinical, or behavioral exemptions), in protected/grooming contact, or small social groups. We follow the recommendations of the guide for the care and use of laboratory animals [2] for minimum required cage sizes (0.40 m^2^–0.74 m^2^ per individual stainless-steel cage). The indoor environment is regulated with a 12:12 h light–dark cycle, maintaining temperatures between 20- and 22 degrees °C and a humidity level of 30–70%. The animals were fed LabDiet 5047 Jumbo primate biscuits twice daily and had ad lib access to water. They were also provided with a perch, a variety of environmental enrichment items, a daily forage board mix, and fresh or dried produce twice weekly.

## 3. Results

In the following section, we provide examples of the review process and their outcomes. These examples illustrate the complexities of this process and highlight the importance of sharing information and viewpoints among all stakeholders to make informed decisions that benefit the individual, the social group, and the CNPRC’s research goals. Each presented case includes the sex and age class (i.e., infant, juvenile, adult, and geriatric), the selection criteria that justified the consideration for permanent relocation from the group, the ensuing discussion, and, finally, the decision.

### 3.1. Manipulating the Social Structure: Instigators Versus Recipients of Aggression

The common feature among the following three cases was the identification of the instigators of aggression and focusing on them as the source of social disruption rather than those who were targeted.

A juvenile female rhesus macaque was presented for evaluation for removal from her group due to reoccurring trauma events, including digit amputations, tail amputation, and gingival laceration. Her most recent trauma occurred one day after she had been discharged back to the group following her previous injury (Section 2.2.1, criteria 4 and 5). The nature and frequency of the trauma events were alarming and required a management decision that would bring an end to these injuries. One suggestion made by the veterinarians was to relocate this animal out of the social group and into an indoor setting, where she would be safe from physical harm inflicted by others. While this proposal would eliminate the likelihood of social trauma, the behavioral management staff voiced concerns about the animal’s normal development in an indoor and more confined setting, as indoor-reared rhesus macaques are approximately ten times more likely to develop motor stereotypic behaviors (reflecting a welfare concern) than their outdoor-reared counterparts [23]. Convincingly, Gottlieb et al. [23] found that being housed in large social groups outdoors provided a buffer from developing motor stereotypic behaviors, as every year spent outdoors decreased the likelihood of expressing these abnormal behaviors by 20%. Supporting these findings, the permanent relocation of adult male rhesus macaques from outdoor social groups into single housing indoors led to the development of depressive-like states (e.g., hunched, withdrawn) [47]. We would like to emphasize that incorporating aspects of behavioral management (i.e., socialization, training, and environmental enrichment) into indoor animals’ routine care is likely to reduce the frequency of abnormal behaviors [48]. Still, the most potent tool to prevent and reduce said behaviors has been suggested to be normal development in complex and species-appropriate social environments [49]. Indeed, large and socially complex groups, similar to those at the CNPRC colony, promote normal development via social learning (e.g., appropriate maternal behavior and other social behaviors) from family members and peers [50]. Therefore, after the discussion, it was decided that the instigators of aggression, including the alpha male, would be removed from the group, whereas the juvenile female would remain in the group following treatment. Since then, this animal has stayed in the group without sustaining additional trauma. The removed alpha male was relocated back into indoor housing and was successfully reunited with his previous pair mate. He remained in continuous full contact with his social partner for three years before being introduced to a new social group. Importantly, this group was newly formed, allowing for the flexibility of introducing another male. The early stage of the group formation, combined with the involvement of the alpha male in multiple conflicts with other animals, ultimately led to the alpha male’s removal. Usually, social introductions of adult males into established groups are risky [51] and, therefore, avoided at the CNPRC.

A second case involved a young adult male rhesus macaque that was presented to the working group following two leg lacerations in two weeks (Section 2.1, criterion 4). As with the previous case, by employing systematic observations, behavioral management staff could identify the individual that instigated the aggression. The instigator was the fourth ranked male at the time of the removal that had been making attempts to climb up the hierarchy. This male was additionally observed challenging the alpha male. Once the information was provided to the working group, the instigator was permanently removed from the social group, and the male victim was successfully discharged back to the group (no trauma was sustained for a year following discharge). The male that was attacked had previously held a higher rank, and after the aggressor was removed, he and other affected males regained their former status. This male remained in the group for an additional two years and was then relocated indoors due to an unrelated social overthrow of the group.

**Figure 5:**
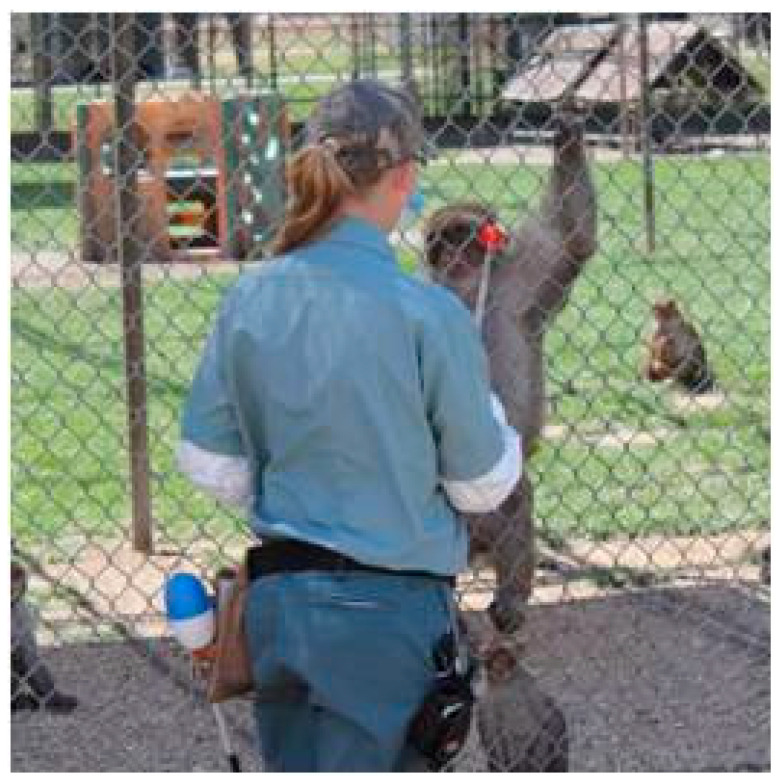
Behavioral management staff train the macaques, using positive reinforcement techniques for a variety of purposes, including shifting.

Finally, a juvenile male rhesus macaque was discussed at a group meeting due to repeated events of social aggression that resulted in multiple injuries to the extremities in six months (Section 2.1, criteria 2 and 5). The working group’s discussion highlighted that the aggressor was the alpha female in the first three incidents. Behavioral observations of the group revealed ambiguity in ranks and a lack of confidence in social status involving interactions of the alpha male with both the alpha female and younger females. Subsequently, the alpha female was removed from the group (as with the first case, this was a newly formed group, which enabled the behavioral management staff to replace her). Further observations showed that the last incident involved the alpha male as the aggressor and occurred in conjunction with routine daily cleaning of the enclosure. Husbandry procedures, including frequent ones, can be a source of stress to the animals. For example, indoor cage cleaning and changing, which occurs every two weeks, was reported to induce the expression of anxiety-related behaviors in research rhesus monkeys [52], and similar outcomes were reported for some experimental procedures [53]. NHPs housed in standard stainless-steel cages are less able to cope with stressors they encounter by employing species-typical behaviors than their counterparts housed in much larger and more structurally complex enclosures. For example, the ability to move away from perceived threats (e.g., human activity, noise, and hosing) is clearly curtailed in smaller cages as the animals’ degree of control and choice over the environment is minimal [54]. We emphasize that there is a plethora of refinements to both husbandry and research procedures (e.g., positive reinforcement training and visual barriers) that can help the animals be in better control of their environment and thus better cope with the stressors they present (e.g., [55]). Indeed, successful coping is the main goal of behavioral management programs since, as Broom [56] suggested, “the welfare of an individual is its state as regards its attempts to cope with its environment”. Namely, an animal that can successfully cope with its environment likely experiences superior welfare over an unsuccessful animal. In response to the information, the behavioral management staff created and implemented a training plan (based on the principles of positive reinforcement training techniques) to shift the alpha male to a different location within the enclosure before potentially stressful events to prevent further trauma to younger individuals (Figure 5). The animal was reliably trained to shift within a week, and, due to his rank, no interferences in training by other group members were noted.

### 3.2. Manipulating the Environment: Providing Social Support and Environmental Enrichment

In the previous section, we described the removal of specific individuals to render the social environment less hostile for some monkeys. The following section focuses on cases in which certain individuals, environmental enrichment items, or both were added or removed before the discharge of the recipients of aggression to enable a more supportive environment. The first case involved an adult beta female that was presented to the working group for general trauma (Section 2.1, criterion 1). Upon investigation by the working group, it was found that the aggressive event occurred while the alpha female was temporarily absent from the group for a medical procedure. The alpha female was known to provide social support to the beta female, and it was thus decided to discharge the beta female once the alpha female had returned. The beta female was able to successfully return to the group and remain in it without sustaining additional trauma.

In another case, a juvenile male was presented to the working group after requiring multiple digit amputations in a single trauma event (Section 2.1, criteria 1 and 2). The young age of this male and a history of SIB raised concerns about his prospects of developing normally in an indoor environment. Therefore, rather than relocating this animal into an indoor environment, the working group proactively manipulated the group’s social structure, discharging this male back to his group only after a new alpha male was introduced and two adult females were permanently removed and relocated indoors, where they awaited project reassignment.

Lastly, an adult male was hospitalized multiple times in less than twelve months due to digit and tail trauma (Section 2.1, criteria 2 and 5). The male was part of a group with relatively high rates of digit trauma that was additionally evaluated in a separate study examining the impact of long-lasting produce on reduction in social trauma [45,46]. Attempting to maintain the integrity of the ongoing research, the working group decided to discharge this male back to his social group in conjunction with providing novel, long-lasting enrichment aimed specifically at reduction in digit trauma. Unfortunately, this male had one additional incident of trauma three months following his discharge and was subsequently relocated indoors.

### 3.3. Challenges with Coping Indoors

Animals may be required to be removed from their social group for a variety of reasons. Thus, in addition to social reasons, NHPs may be removed for research project screening and assignment, medical concerns, and shipment to other facilities. The indoor environment differs markedly from the outdoor one and poses unique challenges for the animals. In this section, we will describe three cases of individual macaques that needed to be removed from their group and appeared to struggle to cope indoors.

The first case involves an adult female macaque that was removed from the group for research project screening. Since the removal was unrelated to social concerns, the animal was not initially brought up by the working group. However, once indoors, she was singly housed and began exhibiting SIB, suggesting unsuccessful attempts to reduce arousal by employing species-typical behaviors [57]. The indoor environment at the CNPRC differs from the outdoor one in many aspects. First, the space available for indoor animals is significantly smaller than outdoors. Most of the indoor population is housed in standard cages that fit regulatory requirements [1,2]. For example, an animal such as this female that weighs less than 10 kg is required to be housed in a cage with a minimum floor space of 0.4 square meters and a height of 76.2 cm. The relatively confined space is less positively stimulating, but, perhaps more importantly, it reduces the animal’s sense of control over the environment, which is central to their welfare [58]. Here, the project screening process involved accessing the animals several times to collect samples and perform pre-project physical exams, which can be stressful and result in the development of abnormal behaviors [59]. This case was then discussed among behavioral management and veterinary staff, and, out of concern for this female’s welfare, she was consequently dropped from the research project and returned successfully to her group, where she has not exhibited SIB since.

The second case also involves an adult female rhesus macaque. This female was out of the group for clinical treatment following incurring social trauma. Upon discharge back to the group, the behavioral management staff observed her being attacked by several group members and decided to relocate her permanently indoors (Section 2.1, criterion 4). Once indoors, however, animal care and veterinary staff noticed a weight loss trend, which was used as a welfare indicator [60]. Indeed, despite being pair-housed in continuous full contact with a compatible partner, this female lost 30% of her body in eleven months. The veterinary staff, therefore, recommended including the animal in a future group formation. The behavioral management staff included this animal when a new social group was successfully formed, and the animal regained its original weight in four months.

The third case regarded a geriatric female rhesus macaque brought indoors during a social overthrow in her group. Similar to the previous case, this animal also lost weight while housed indoors, which may suggest unsuccessful coping and poor welfare [60]. The veterinarians requested to include her in an upcoming group formation. Unfortunately, the animal was rejected from the new group and had to be relocated once again. However, rather than housing her in an environment in which she struggled before, the behavioral management staff successfully integrated her into a small social group indoors as part of a research project. Despite being housed indoors, the social complexity of the group likely enabled the animal to enlist her group members to buffer the stressors she encountered, as evidenced by the stability of her weight.

### 3.4. Social Stability Concerns Related to Adult Male-to-Female Ratio

The working group evaluated a case of an adult male rhesus macaque following several injuries to his limbs in under a year (Section 2.1, criterion 5). While the concern for this individual was obvious to all, the working group identified additional problems at the group level. Specifically, the adult male-to-adult female ratio in the group was already skewed (too few males to females) to a degree associated with social instability and increased social trauma. Our behavioral management practices aim for a ratio of two to three adult females to one adult male [61], similar to the sex ratio observed in wild populations of rhesus macaques [62]. Males serve an important regulating role in rhesus societies. Males can increase group stability through conflict policing, whereby they employ their high social power to stop other group members from fighting [63,64]. Therefore, in groups with a lower male-to-female ratio, one can expect higher intra-group aggression rates. In this case, the working group needed to weigh the costs and benefits of removing this male and ameliorating potentially socially destabilizing factors. The decision was to discharge the animal back to his group, carefully monitor his return, and present the case again if the animal sustained additional trauma in the next six months. Unfortunately, this male was injured again about a month following his return to the group and was subsequently permanently removed from his group. No increase in trauma was noted following the male’s removal, suggesting that his regulatory effect was mild. In the future, a priori characterization of the males’ social power and policing capabilities could assist the working group in reaching informed decisions (e.g., in this case, knowing that the male did not have significant ability to intervene and stop fights might have resulted in earlier removal) [64]. Using social networks (Figure 1) is one of the main tools that enables behavioral management staff to characterize an individual’s social significance.

## 4. Discussion

A complex social structure and environmental conditions are critical factors influencing the health and welfare of rhesus macaques in captivity. The CNPRC employs a detailed and systematic approach to managing the welfare of these animals, taking into account both individual and group dynamics. Specific criteria guide the review process for potentially removing individuals from their social groups and involve input from behavioral management experts, principal investigators who conduct research in the outdoor environment, veterinarians, and animal care staff.

It is evident that while social living provides significant benefits, it also comes with challenges, such as competition and aggression. Therefore, careful management is needed to ensure that animals have access to resources (e.g., food, water, enrichment) and are not the target of repeated aggression. The examples provided highlight the nuanced decision-making process involved in addressing aggression and trauma within these groups. Decisions are made not only to protect the individual animal but also to maintain the overall stability and health of the social group.

Key strategies include the removal of aggressive individuals, provision of enrichment items, and enhanced monitoring to prevent social collapse. However, as we have demonstrated, interventions may fall short of achieving their goals, and this highlights the need for contingency plans. Furthermore, while the criteria for identifying animals for potential relocation (Section 2.2.1) and whether they should be removed (Section 2.2.2) are clear and precise, the discussion that ensues factors them in, along with the various points of view brought about by all parties involved. Indeed, the working group’s approach underscores the importance of a multi-faceted strategy that considers clinical and behavioral indicators and their interplay when making management decisions. The working group’s main concern is the welfare of the NHP colony, which includes the animal being discussed but also other animals that may be affected by the decision (e.g., those who may experience an increased trauma rate following the removal of an effective regulator). The concern for the welfare of the CNPRC animals dictates the priority given to the implementation of each case’s decision. When an intervention has the potential to influence study outcomes, the PI is brought into the conversation to facilitate and coordinate the decision implementation with minimal negative impact on the research. We have shown that this holistic management is critical in ensuring that all aspects that can influence the welfare of the individual and the group are included in the decision-making process. It is also essential for maintaining a stable and productive breeding and research colony, particularly in light of challenges such as the COVID-19 pandemic, which has underscored the need for a robust and resilient population of NHPs for research purposes.

## Data Availability

The data presented in this study are available on request from the corresponding author.

## References

[1] U (2013). S. Department of Agriculture. Animal Welfare Act and Animal Welfare Regulations (“Blue Book”).

[2] Council N.R. (2011). Guide for the Care and Use of Laboratory Animals.

[3] Cooper E.B., Brent L.J.N., Snyder-Mackler N., Singh M., Sengupta A., Khatiwada S., Malaivijitnond S., Qi Hai Z., Higham J.P. (2022). The rhesus macaque as a success story of the Anthropocene. eLife.

[4] Widdig A., Langos D., Kulik L. (2016). Sex differences in kin bias at maturation: Male rhesus macaques prefer paternal kin prior to natal dispersal. Am. J. Primatol..

[5] Beisner B.A., Jackson M.E., Cameron A.N., McCowan B. (2011). Detecting Instability in Animal Social Networks: Genetic Fragmentation Is Associated with Social Instability in Rhesus Macaques. PLoS ONE.

[6] Maestripieri D., Georgiev A.V. (2016). What cortisol can tell us about the costs of sociality and reproduction among free-ranging rhesus macaque females on Cayo Santiago. Am. J. Primatol..

[7] Henkel S., Lambides A.R., Berger A., Thomsen R., Widdig A. (2015). Rhesus macaques (*Macaca mulatta*) recognize group membership via olfactory cues alone. Behav. Ecol. Sociobiol..

[8] Silk J.B., Gintis H., Bowles S., Boyd R., Fehr E. (2005). The evolution of cooperation in primate groups. Moral Sentiments and Material Interests: The Foundations of Cooperation in Economic Life.

[9] Lehmann J., Korstjens A.H., Dunbar R.I.M. (2007). Group size, grooming and social cohesion in primates. Anim. Behav..

[10] Kempes M.M., Den Heijer E., Korteweg L., Louwerse A.L., Sterck E.H.M. (2009). Socially deprived rhesus macaques fail to reconcile: Do they not attempt or not accept reconciliation?. Anim. Behav..

[11] Coria-Avila G.A., Pfaus J.G., Orihuela A., Domínguez-Oliva A., José-Pérez N., Hernández L.A., Mota-Rojas D. (2022). The Neurobiology of Behavior and Its Applicability for Animal Welfare: A Review. Animals.

[12] Dawkins M.S. (1990). From an animal’s point of view: Motivation, fitness, and animal welfare. Behav. Brain Sci..

[13] Boissy A., Manteuffel G., Jensen M.B., Moe R.O., Spruijt B., Keeling L.J., Winckler C., Forkman B., Dimitrov I., Langbein J. (2007). Assessment of positive emotions in animals to improve their welfare. Physiol. Behav..

[14] Mason G.J., Cooper J., Clarebrough C. (2001). Frustrations of fur-farmed mink. Nature.

[15] Mason G., Clubb R., Latham N., Vickery S. (2007). Why and how should we use environmental enrichment to tackle stereotypic behaviour?. Appl. Anim. Behav. Sci..

[16] Pomerantz O., Meiri S., Terkel J. (2013). Socio-ecological factors correlate with levels of stereotypic behavior in zoo-housed primates. Behav. Process..

[17] Bloomsmith M.A., Hasenau J., Bohm R.P. (2017). Position Statement. J. Am. Assoc. Lab. Anim. Sci..

[18] Coleman K., Timmel G., Prongay K., Baker K.C. (2023). Common husbandry, housing, and animal care practices. Nonhuman Primate Welfare: From History, Science, and Ethics to Practice.

[19] Hannibal D.L., Bliss-Moreau E., Vandeleest J., McCowan B., Capitanio J. (2017). Laboratory rhesus macaque social housing and social changes: Implications for research. Am. J. Primatol..

[20] Beisner B.A., Hannibal D.L., Vandeleest J.J., McCowan B., Robinson L.M., Weiss A. (2023). Sociality, Health, and Welfare in Nonhuman Primates. Nonhu-man Primate Welfare: From History, Science, and Ethics to Practice.

[21] Balasubramaniam K.N., Beisner B.A., Hubbard J.A., Vandeleest J.J., Atwill E.R., McCowan B. (2019). Affiliation and disease risk: Social networks mediate gut microbial transmission among rhesus macaques. Anim. Behav..

[22] Bai H., Capitanio J.P., Miller L.A., Clougherty J.E. (2021). Social status and susceptibility to wildfire smoke among outdoor-housed female rhesus monkeys: A natural experiment. Heliyon.

[23] Gottlieb D.H., Capitanio J.P., McCowan B. (2013). Risk factors for stereotypic behavior and self-biting in rhesus macaques (*Macaca mulatta*): Animal’s history, current environment, and personality. Am. J. Primatol..

[24] Fontenot B.M., Wilkes M.N., Lynch C.S. (2006). Effects of Outdoor Housing on Self-Injurious and Stereotypic Behavior in Adult Male Rhesus Macaques (*Macaca mulatta*). J. Am. Assoc. Lab. Anim. Sci..

[25] Markham A.C., Gesquiere L.R. (2017). Costs and benefits of group living in primates: An energetic perspective. Philos. Trans. R. Soc. Lond. B Biol. Sci..

[26] Reddon A.R., Ruberto T., Reader S.M. (2021). Submission signals in animal groups. Behaviour.

[27] Judge P.G., De Waal F.B.M. (1997). Rhesus monkey behaviour under diverse population densities: Coping with long-term crowding. Anim. Behav..

[28] Honess P.E., Marin C.M. (2006). Enrichment and aggression in primates. Neurosci. Biobehav. Rev..

[29] Chamove A.S., Anderson J.R., Morgan-Jones S.C., Jones S.P. (1982). Deep woodchip litter: Hygiene, feeding, and behavioral enhancement in eight primate species. Int. J. Study Anim. Probl..

[30] McCowan B., Beisner B., Hannibal D. (2018). Social management of laboratory rhesus macaques housed in large groups using a network approach: A review. Behav. Process..

[31] Bernstein I.S., Gordon T.P. (1974). The Function of Aggression in Primate Societies: Uncontrolled aggression may threaten human survival, but aggression may be vital to the establishment and regulation of primate societies and sociality. Am. Sci..

[32] Judge P.G., de Waal F.B.M. (1993). Conflict avoidance among rhesus monkeys: Coping with short-term crowding. Anim. Behav..

[33] Beisner B.A., McCowan B. (2013). Policing in Nonhuman Primates: Partial Interventions Serve a Prosocial Conflict Management Function in Rhesus Macaques. PLoS ONE.

[34] Flack J.C., de Waal F.B.M., Krakauer D.C. (2005). Social Structure, Robustness, and Policing Cost in a Cognitively Sophisticated Species. Am. Nat..

[35] Oates-O’Brien R.S., Farver T.B., Anderson-Vicino K.C., McCowan B., Lerche N.W. (2010). Predictors of matrilineal overthrows in large captive breeding groups of rhesus macaques (*Macaca mulatta*). J. Am. Assoc. Lab Anim. Sci..

[36] Wooddell L.J., Kaburu S.S., Rosenberg K.L., Meyer J.S., Suomi S.J., Dettmer A.M. (2016). Matrilineal Behavioral and Physiological Changes following the Death of a Non-Alpha Matriarch in Rhesus Macaques (*Macaca mulatta*). PLoS ONE.

[37] Dettmer A.M., Woodward R.A., Suomi S.J. (2015). Reproductive consequences of a matrilineal overthrow in rhesus monkeys. Am. J. Primatol..

[38] Wenyuan Q., Yongzu Z., Manry D., Southwick C.H. (1993). Rhesus monkeys (*Macaca mulatta*) in the Taihang mountains, Jiyuan county, Henan, China. Int. J. Primatol..

[39] Albrecht L., Bishop E., Jay B., Lafoux B., Minoves M., Passaes C. (2021). COVID-19 Research: Lessons from Non-Human Primate Models. Vaccines.

[40] Toufexis D., Rivarola M.A., Lara H., Viau V. (2014). Stress and the reproductive axis. J. Neuroendocrinol..

[41] Fernandez-Novo A., Pérez-Garnelo S.S., Villagrá A., Pérez-Villalobos N., Astiz S. (2020). The Effect of Stress on Reproduction and Reproductive Technologies in Beef Cattle-A Review. Animals.

[42] Lemoine S., Preis A., Samuni L., Boesch C., Crockford C., Wittig R.M. (2020). Between-Group Competition Impacts Reproductive Success in Wild Chimpanzees. Curr Biol..

[43] Kaurivi Y.B., Laven R., Parkinson T., Hickson R., Stafford K. (2020). Effect of Animal Welfare on the Reproductive Performance of Extensive Pasture-Based Beef Cows in New Zealand. Vet. Sci..

[44] Cronin K.A., West V., Ross S.R. (2016). Investigating the relationship between welfare and rearing young in captive chimpanzees (*Pan troglodytes*). Appl. Anim. Behav. Sci..

[45] Wooddell L.J., Beisner B., Hannibal D.L., Nathman A.C., McCowan B. (2019). Increased produce enrichment reduces trauma in socially-housed captive rhesus macaques (Macaca mulatta). Am. J. Primatol..

[46] McCowan B., Vandeleest J., Balasubramaniam K., Hsieh F., Nathman A., Beisner B. (2022). Measuring dominance certainty and assessing its impact on individual and societal health in a nonhuman primate model: A network approach. Philos. Trans. R. Soc. B.

[47] Hennessy M.B., McCowan B., Jiang J., Capitanio J.P. (2014). Depressive-like behavioral response of adult male rhesus monkeys during routine animal husbandry procedure. Front. Behav. Neurosci..

[48] Oppler S.H., Palmer S.D., Phu S.N., Graham M.L. (2024). The Role of Behavioral Management in Enhancing Clinical Care and Efficiency, Minimizing Social Disruption, and Promoting Welfare in Captive Primates. Vet. Sci..

[49] Lutz C.K., Coleman K., Hopper L.M., Novak M.A., Perlman J.E., Pomerantz O. (2022). Nonhuman primate abnormal behavior: Etiology, assessment, and treatment. Am. J. Primatol..

[50] Rox A., Waasdorp S., Sterck E.H.M., Langermans J.A.M., Louwerse A.L. (2022). Multigenerational Social Housing and Group-Rearing Enhance Female Reproductive Success in Captive Rhesus Macaques (Macaca mulatta). Biology.

[51] Bailey K.L., Young L.A., Long C.E., Remillard C.M., Moss S.E., Meeker T.L., Bloomsmith M.A. (2021). Use of Introduction Enclosures to Integrate Multimale Cohorts into Groups of Female Rhesus Macaques (Macaca mulatta). J. Am. Assoc. Lab. Anim. Sci..

[52] Pomerantz O., Nyandwi S., Baker K. (2018). Providing feeding enrichment by hand mitigates anxiety among laboratory-housed rhesus macaques (*Macaca mulatta*). In *American Journal of Primatology*; Wiley: Hoboken, NJ, USA, 2018.mulatta). American Journal of Primatology.

[53] Pfefferle D., Plümer S., Burchardt L., Treue S., Gail A. (2018). Assessment of stress responses in rhesus macaques (*Macaca mulatta*) to daily routine procedures in system neuroscience based on salivary cortisol concentrations. PLoS ONE.

[54] Morgan K.N., Tromborg C.T. (2007). Sources of stress in captivity. Appl. Anim. Behav. Sci..

[55] Jennings M., Prescott M.J., Buchanan-Smith H.M., Gamble M.R., Gore M., Hawkins P., Hubrecht R., Hudson S., Jennings M., Members of the Joint Working Group on Refinement (Primates) (2009). Refinements in husbandry, care and common procedures for non-human primates: Ninth report of the BVAAWF/FRAME/RSPCA/UFAW Joint Working Group on Refinement. Lab. Anim..

[56] Broom D.M. (2011). A History of Animal Welfare Science. Acta Biotheor..

[57] Novak M.A. (2003). Self-injurious behavior in rhesus monkeys: New insights into its etiology, physiology, and treatment. Am. J. Primatol..

[58] Bettinger T.L., Leighty K.A., Daneault R.B., Richards E.A., Bielitzki J.T. (2017). Behavioral management: The environment and animal welfare. Handbook of Primate Behavioral Management.

[59] Novak M.A., Meyer J.S. (2021). Abnormal behavior of animals in research settings. Behavioral Biology of Laboratory Animals.

[60] Prescott M.J., Leach M.C., Truelove M.A. (2022). Harmonisation of welfare indicators for macaques and marmosets used or bred for research. F1000Research.

[61] Oldt R.F., Beisner B., Cameron A., Pomerantz O., Kanthaswamy S. (2023). Pedigree Data from Six Rhesus Macaque (Macaca mulatta) Matrilines at the California National Primate Research Center Indicate Inbreeding and Loss of Genetic Variation. J. Am. Assoc. Lab. Anim. Sci..

[62] Hernández-Pacheco R., Rawlins R.G., Kessler M.J., Williams L.E., Ruiz-Maldonado T.M., González-Martínez J., Ruiz-Lambides A.V., Sabat A.M. (2013). Demographic variability and density-dependent dynamics of a free-ranging rhesus macaque population. Am. J. Primatol..

[63] Beisner B.A., Hannibal D.L., Finn K.R., Fushing H., McCowan B. (2016). Social power, conflict policing, and the role of subordination signals in rhesus macaque society. Am. J. Phys. Anthropol..

[64] Pritchard A.J., Beisner B.A., Nathman A., McCowan B. (2024). Social stability via management of natal males in captive rhesus macaques (*Macaca mulatta*). J. Appl. Anim. Welf. Sci..

